# Supplementary material for: Learning the histone codes with large genomic windows and three-dimensional chromatin interactions using transformer
Source: Nat Commun. 2022 Nov 5;13:6678. doi: 10.1038/s41467-022-34152-5 (PMC9637148; doi:10.1038/s41467-022-34152-5)
Supplement: Supplementary file 1 — Supplementary Information [file 41467_2022_34152_MOESM1_ESM.pdf]

Learning the histone codes with large genomic windows and three-dimensional chromatin interactions using transformer

Lee *et al.*

Supplementary Note 1. Difference between Embedding transformer and Pairwise interaction transformer

The reason why we decided to give these two substructures (Embedding and Pairwise Interaction transformers) separate names can be explained by the following two aspects of differences between them: (1) Difference in the type of attention operation used, and (2) difference in learning semantics.

Difference in the type of attention operation

The critical difference between Embedding and Pairwise Interaction transformers is that the former is essentially based on self-attention operation, and the latter is based on encoder-decoder attention. Here, we explain the differences between those two variants of attention operations to emphasize the difference between Embedding and Pairwise Interaction transformers. The core operation for all the three types of transformers in Chromoformer is the Query-Key-Value attention (denoted as red boxes labeled with 'Multi-Head Attention' in Figure 1c-e in the main text). Briefly, Query-Key-Value attention produces the updated version of query embeddings as the weighted sum of Value embeddings. Here, the weights are determined through the computation of affinities between Query and Key embeddings. The critical difference between self-attention and encoder-decoder attention is that self-attention generates both Query and Key embeddings from a single sequence (or set of vectors), while encoder-decoder attention generates Query and Key embeddings separately from two different sequences. Therefore, self-attention measures the 'affinities' between two positions within a single sequence, while encoder-decoder attention measures the affinities between two positions from two independent sequences. This apparently small difference results in a crucial difference in the semantics of Chromoformer learning, which is discussed in the following.

Semantic difference

Since the core operation within the Embedding transformer and Pairwise Interaction transformer is different, what they are designed to learn is also different. An Embedding transformer only takes a core promoter feature as an input, and is trained to capture the intra-dependencies of HM configurations at different positions within the given core promoter. On the other hand, a Pairwise Interaction transformer takes a pair of a core promoter and a corresponding pCRE as input, and learns the pairwise dependencies between the two positions in the core promoter and the pCRE.

Supplementary Method 1. Computation of normalized interaction frequencies

In this study, normalized interaction frequencies were used instead of raw interaction frequencies because there are some technical biases in raw interaction frequencies that hampers the direct interpretation of those values. First, due to the regional preference of a sequencing experiment, restriction and alignment methods, the coverage or mappability of Hi-C sequencing reads throughout the genome is not uniform. This is exacerbated in pcHi-C experiments since the fragment containing the promoter is significantly high due to promoter-enrichment procedure (For example, the raw coverage of promoter fragment is about 14.4 times higher than non-promoter fragments for H1 pcHi-C data used in this study). Thus, the frequencies of promoter-promoter interactions would be more exaggerated than the true amount of interactions between them. Next, the random Brownian motion of DNA polymer results in higher frequency of non-biological interactions between the two fragments at closer linear distance along the genome. This distance bias should be corrected because otherwise the results would erroneously favor interactions at close distances and ignore long-range biological contacts such as promoter-enhancer interactions.

Regarding the two aforementioned biases, normalized interaction frequencies were obtained by statistically correcting them. We note that the formulation of normalization procedure described below is adopted from the R package covNorm v1.1.0[1], since the pcHi-C data in 3div employs it. First, the coverage bias is corrected by fitting a negative binomial regression model for raw ligation frequencies between two fragments using individual coverage values. Formally, the raw interaction frequency (i.e., read ligation frequencies) between two DNA fragments i and j, Y_{ij}, is normalized using the coverages C_i and C_j as follows. Using values of Y_{ij}, the expected interaction frequency u_{ij} is fitted by negative binomial regression model $\log(u_{ij}) = \beta_0 + \beta_1 C_i + \beta_2 C_j$. Then, the normalized interaction frequency R_{ij} is obtained by taking residual $R_{ij} = Y_{ij}/\exp(\beta_0 + \beta_1 C_i + \beta_2 C_j)$.

Subsequently, distance bias is corrected in a similar manner. Given the linear distance between two genomic fragments i and j, D_{ij}, the expected ligation frequency was fitted by negative binomial regression model $\log(u_{ij}) = \beta_0 + \beta_1 D_{ij}$. When $D_{ij} = d$, the expected ligation frequency is given by $E_d = \exp(\beta_0 + \beta_1 d)$. Therefore, the distance-dependent signal can be removed by taking residual $(R_{ij} + \mathrm{avg}(R_{ij}))/(E_d + \mathrm{avg}(R_{ij}))$, where $\mathrm{avg}(R_{ij})$ is a global average value of R_{ij}'s.

Supplementary Table 1. **ENCODE file accessions of PRC1 and PRC2 subunit ChIP-seq peaks.**

Target	ENCODE file accession
EZH2	ENCFF798ICZ, ENCFF833UQN, ENCFF414CAB, ENCFF782TOJ
SUZ12	ENCFF225AMM, ENCFF297ZWL, ENCFF521PXA
RNF2	ENCFF352IAI, ENCFF147QRM, ENCFF241UKW
CBX8	ENCFF483UZG, ENCFF891TAW, ENCFF756MTY

Supplementary Table 2. **ENCODE file accessions of raw ChIP-seq reads for ES-Bruce4 mouse embryonic stem cell.**

Target	ENCODE file accession
H3K4me1	ENCFF001KEF
H3K4me3	ENCFF001KER, ENCFF001KEQ
H3K9me3	ENCFF001KDP, ENCFF001KDM
H3K27me3	ENCFF001KED, ENCFF001KEC
H3K36me3	ENCFF001KEE, ENCFF001KEI
H3K27ac	ENCFF001KDQ, ENCFF001KDO
H3K9ac	ENCFF001KDK, ENCFF001KDL

Supplementary Table 3. **ENCODE file accessions of raw CTCF ChIP-seq reads.**

Epigenome ID	Cell type description	ENCODE file accession
E003	H1 cells	ENCFF000ONR, ENCFF000OOF
E007	H1 derived neuronal progenitor cultured cells	ENCFF342XVP, ENCFF997NPD, ENCFF717KPM
E114	A549 EtOH 0.02pct lung carcinoma	ENCFF000AHW, ENCFF000AHX
E116	GM12878 lymphoblastoid	ENCFF000VUW, ENCFF000VUU
E118	HepG2 hepatocellular carcinoma	ENCFF186EUH, ENCFF023MCP

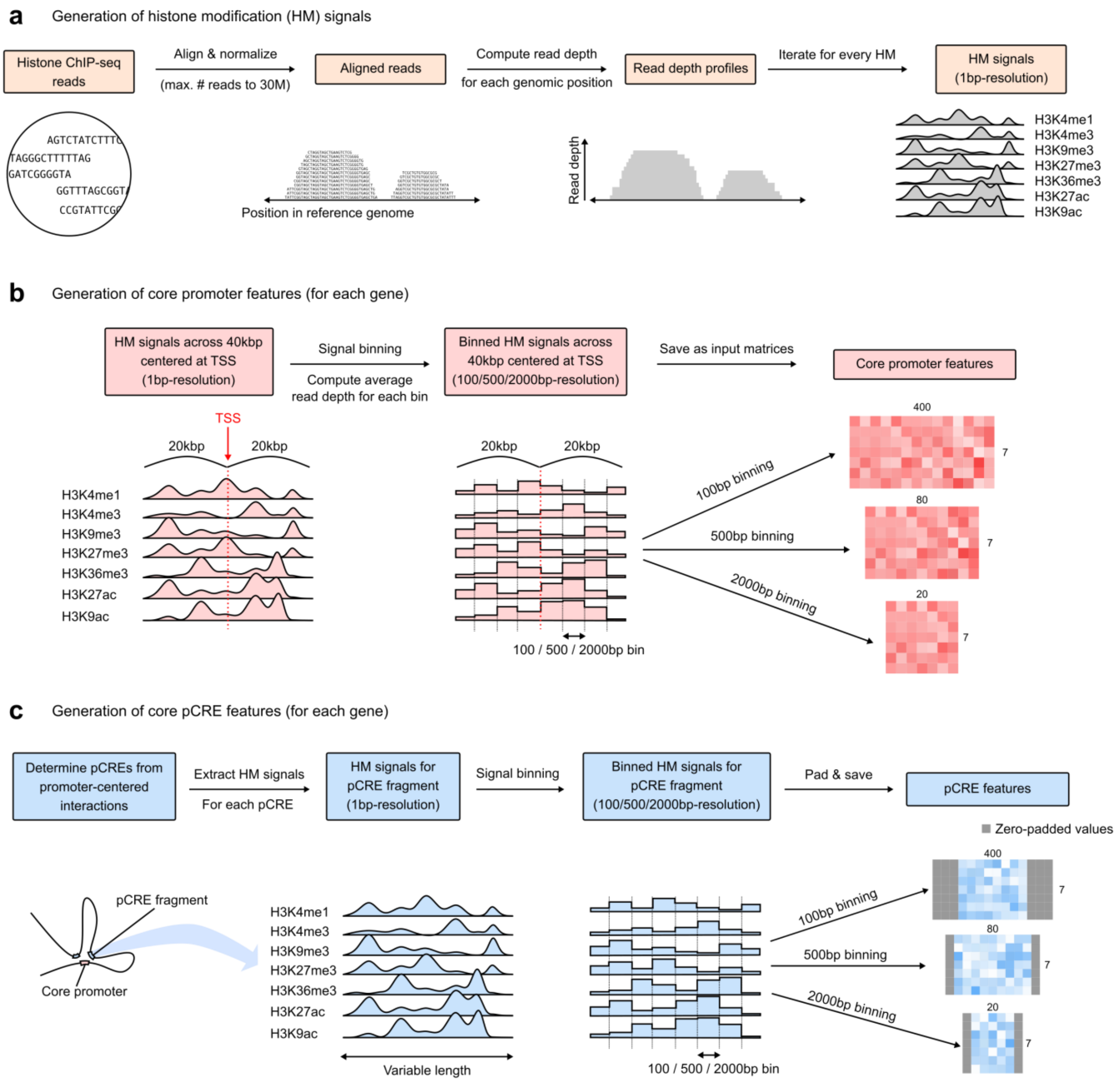

Supplementary Figure 1. Input feature generation procedures. (a) Preparation of histone modification signals. (b) Generation of core promoter features. (c) Generation of core pCRE features.

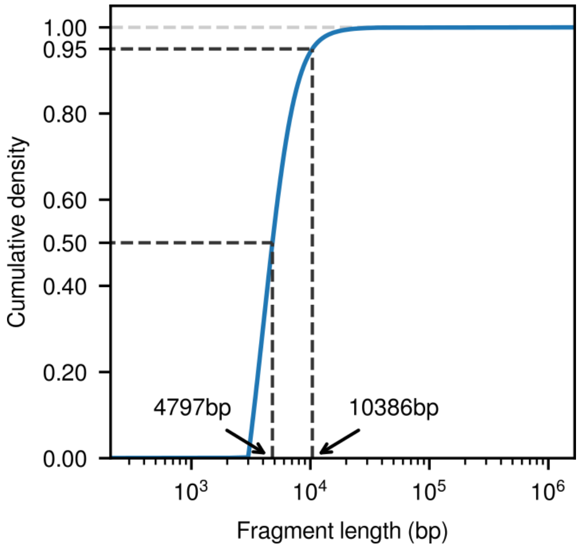

Supplementary Figure 2. **Distribution of HindIII fragment length from the pcHi-C dataset used in this study.**

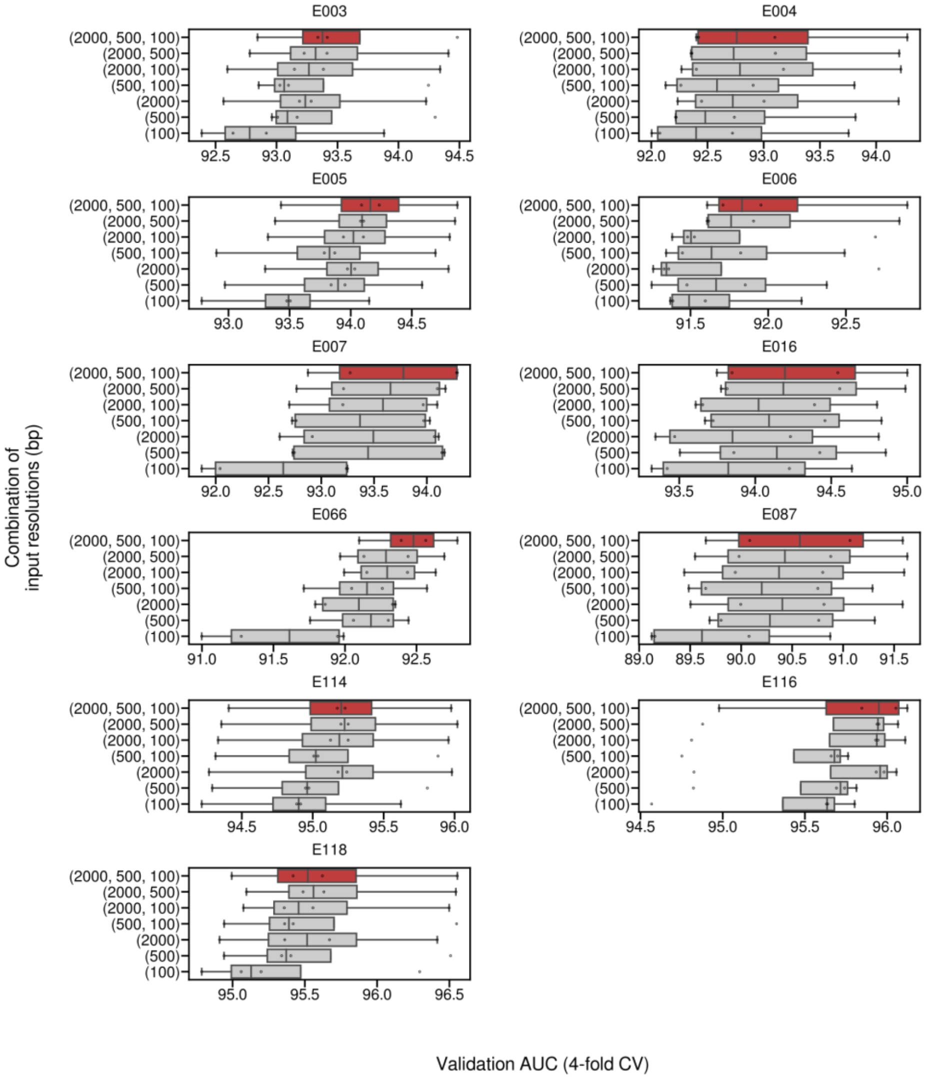

Supplementary Figure 3. Cross-validation (n=4) performances of Chromoformer-clf models when different combinations of resolutions were used. In the boxplot, the center line denotes the median, upper and lower box limits denote upper and lower quartiles, and whiskers denote 1.5× interquartile range. Source data are provided as a Source Data file.

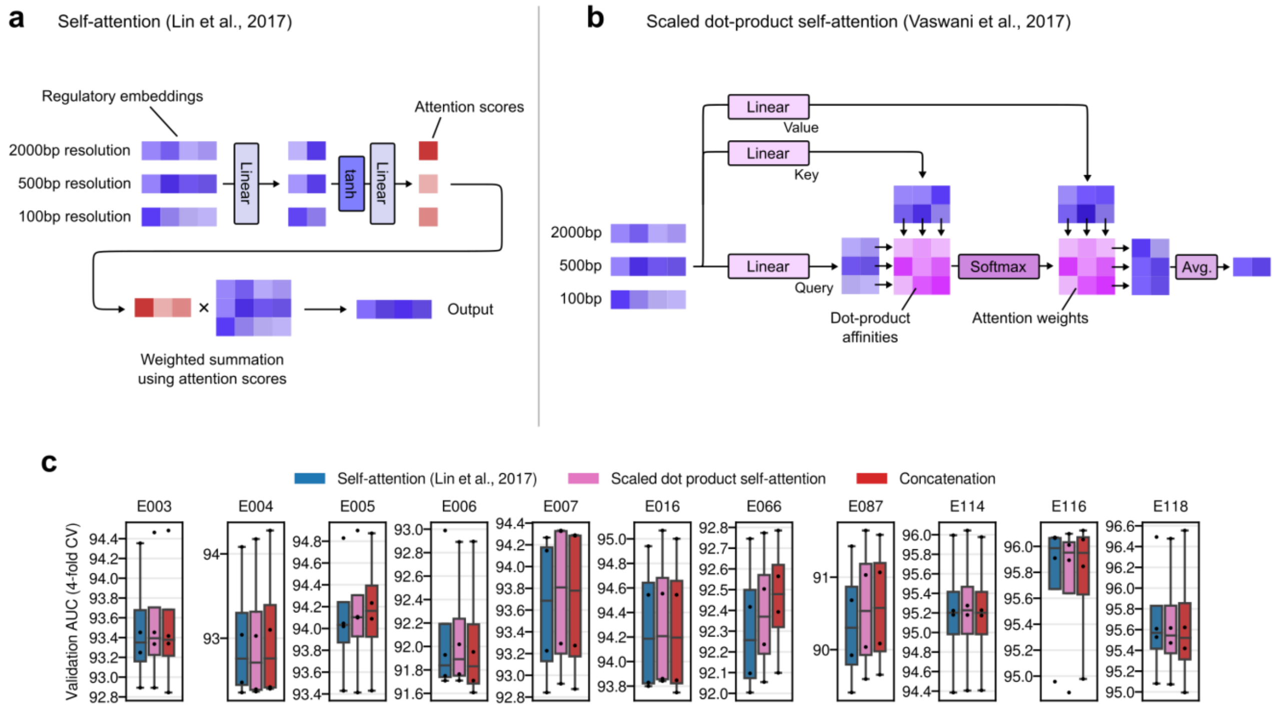

Supplementary Figure 4. Chromoformer-clf model performances when self-attention-based aggregation of regulatory embeddings was used instead of concatenation. (a) Schematic illustration of self-attention operation proposed by Lin *et al*. (b) Schematic illustraction of scaled dot-product attention proposed by Vaswani *et al*. (c) Cross-validation (n=4) performances of Chromoformer-clf models. In the boxplot, the center line denotes the median, upper and lower box limits denote upper and lower quartiles, and whiskers denote 1.5× interquartile range. Source data are provided as a Source Data file.

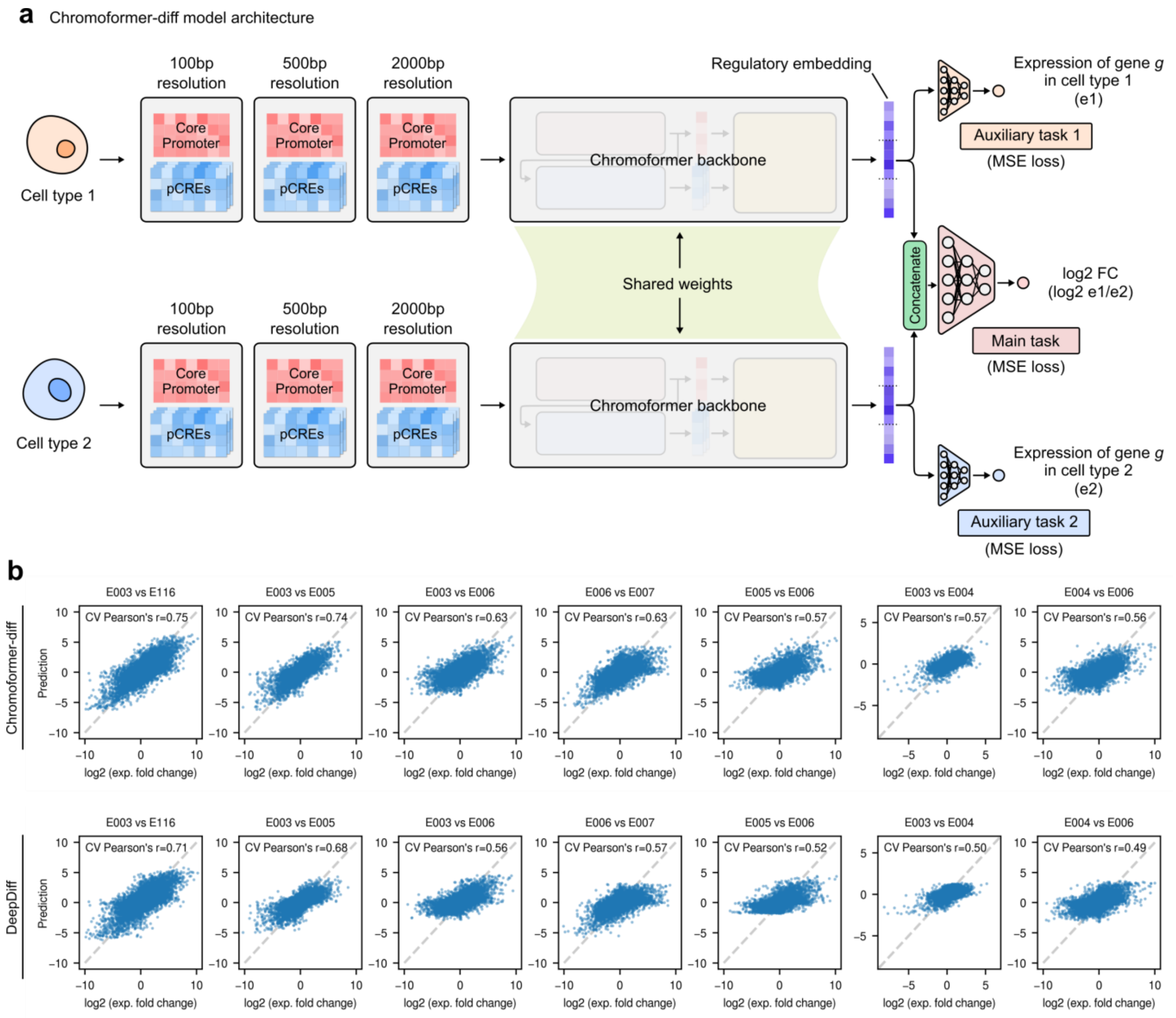

Supplementary Figure 5. Chromoformer-diff model architecture and performance. (a) Schematic illustration of Chromoformer-diff model architecture. (b) Examples of Chromoformer-diff predictions for log2 (expression fold change). Note that 4-fold cross-validation predictions were pooled into a single plot. Source data are provided as a Source Data file.

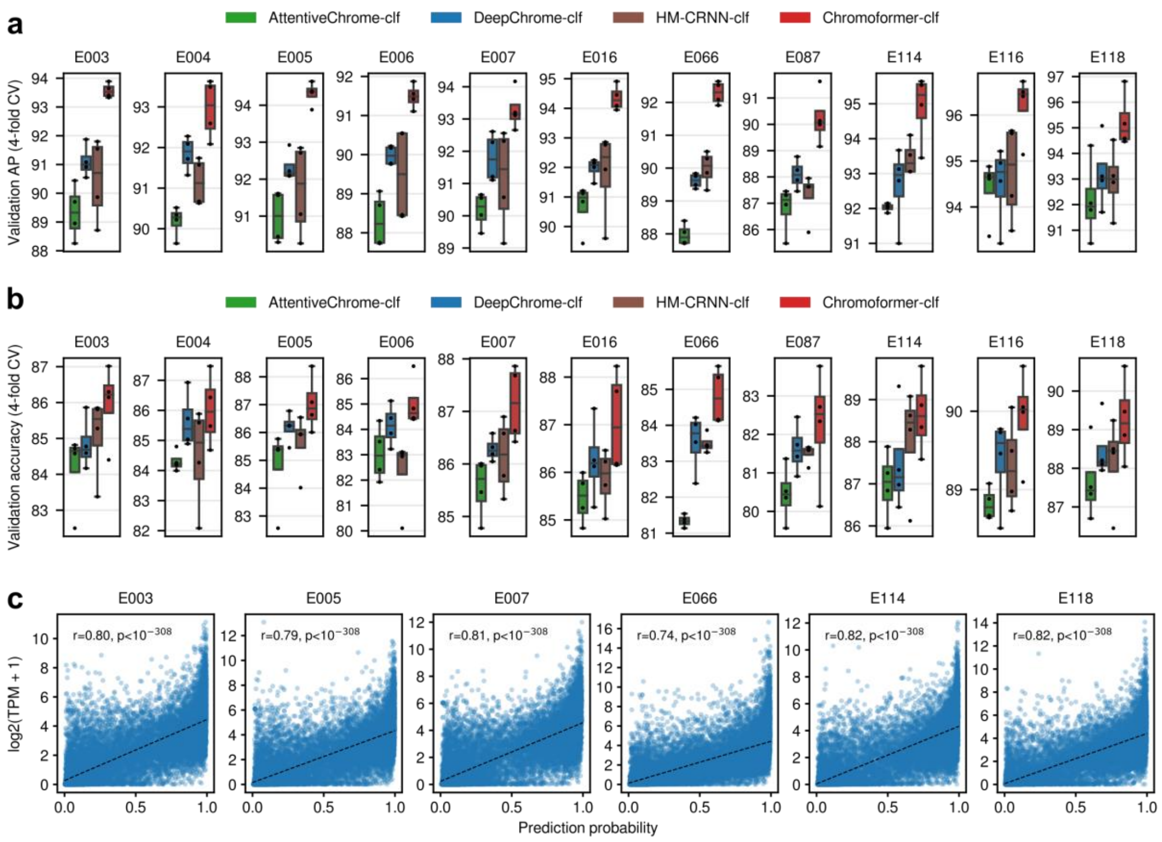

Supplementary Figure 6. Chromoformer-clf model performance. Comparison of cross-validation (n=4) (a) average precisions and (b) accuracies between benchmark models and Chromoformer-clf. In the boxplot, the center line denotes the median, upper and lower box limits denote upper and lower quartiles, and whiskers denote 1.5× interquartile range. (c) The prediction probability was highly correlated with the actual expression levels (Pearson's correlation coefficient (r) > 0.7, all $p < 10^{-308}$ for correlation coefficients). AP, average precision. Source data are provided as a Source Data file.

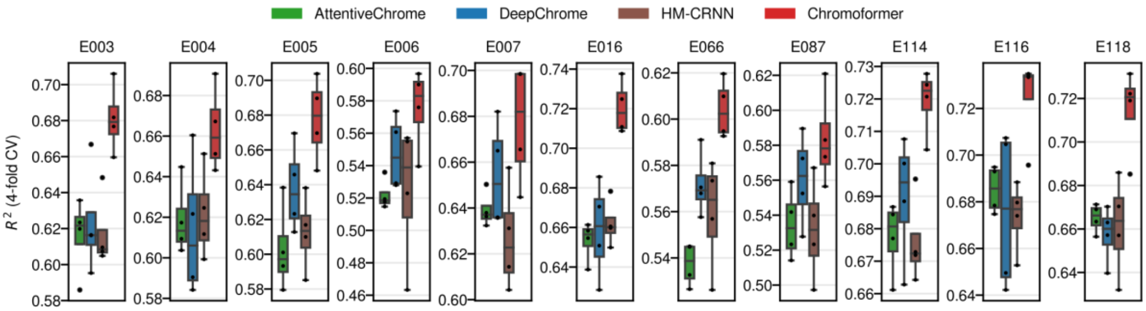

Supplementary Figure 7. Chromoformer-reg model performance. Cross-validation (n=4) performances of Chromoformer-reg models in terms of R^2 value. In the boxplot, the center line denotes the median, upper and lower box limits denote upper and lower quartiles, and whiskers denote 1.5× interquartile range.

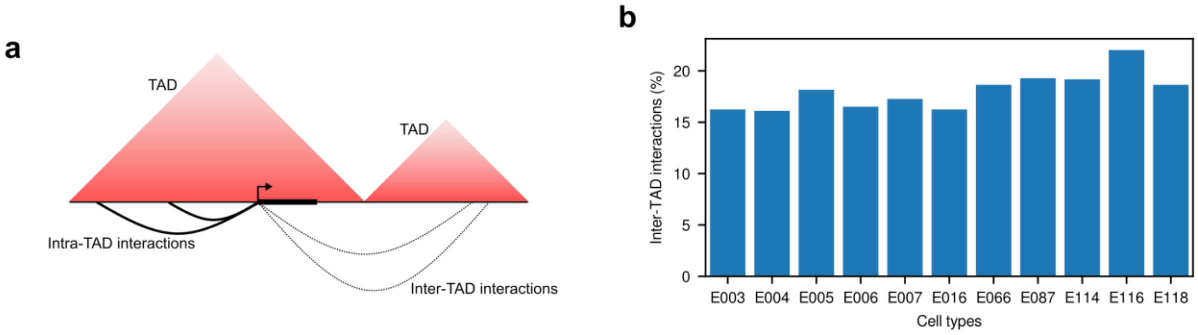

c

Genes with *cis*-regulatory interactions (Δ average validation AUC)

EID	E003	E004	E005	E006	E007	E016	E066	E087	E114	E116	E118
Without inter-TAD	-0.02	+0.02	-0.01	-0.06	+0.01	-0.06	-0.02	+0.01	-0.05	-0.06	-0.02
Without intra-TAD	-0.08	-0.01	-0.08	-0.14	-0.02	-0.13	-0.08	-0.14	-0.08	-0.04	-0.04

Genes without *cis*-regulatory interactions (Δ average validation AUC)

EID	E003	E004	E005	E006	E007	E016	E066	E087	E114	E116	E118
Without inter-TAD	-0.02	-0.13	+0.08	+0.16	-0.34	-0.19	+0.16	+0.08	+0.13	+0.57	+0.04
Without intra-TAD	+0.30	+0.19	+0.22	+0.09	-0.24	+0.02	+0.51	+0.38	+0.19	+0.81	+0.20

Supplementary Figure 8. Contribution of inter- and intra-TAD chromatin interactions in Chromoformer training. (a) Schematic illustration of inter- and intra-TAD chromatin interactions. (b) Proportion of inter-TAD promoter-pCRE interactions used in Chromoformer-clf training for each cell type. (c) Performance differences of Chromoformer-clf models when inter- and intra-TAD interactions were excluded from training. Average validation AUC scores were separately measured for genes with and without at least one *cis*-regulatory interaction. Source data are provided as a Source Data file.

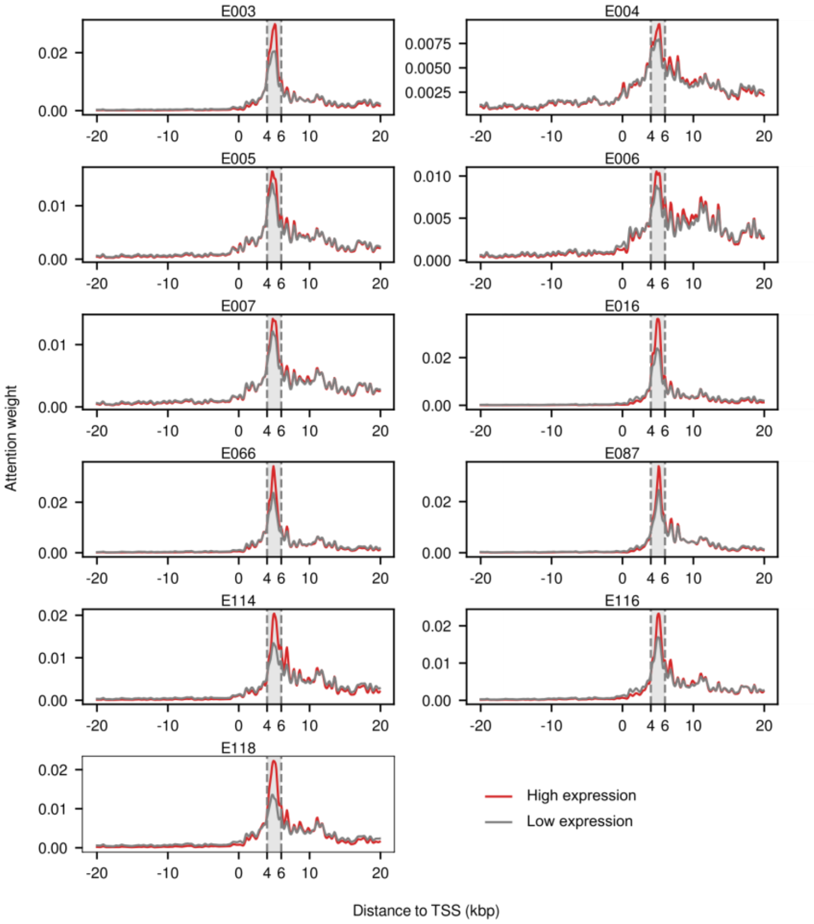

Supplementary Figure 9. Across-cell type consistency of self-attention weights learned by the Embedding transformer of Chromoformer-clf. Epigenome ID denoting the corresponding cell type is shown above each plot. Source data are provided as a Source Data file.

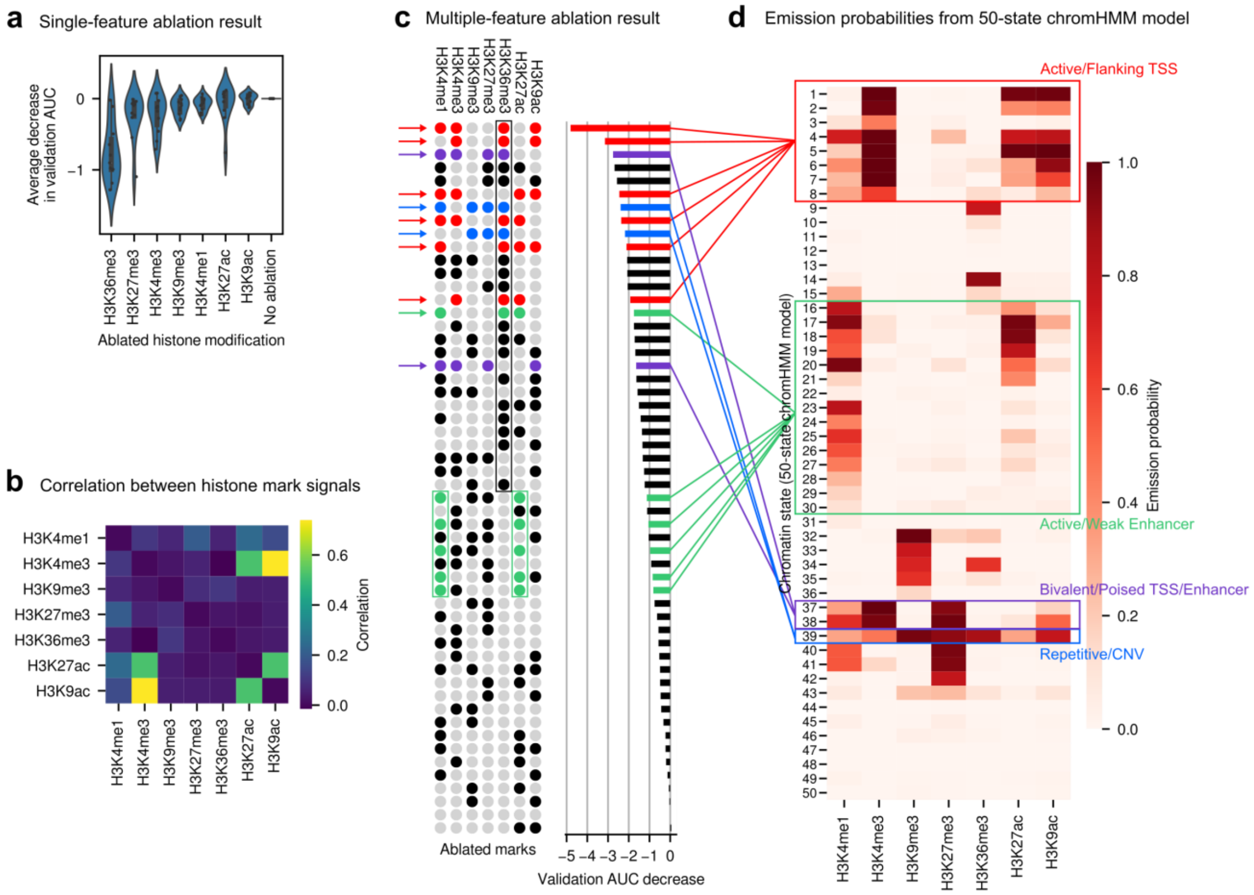

Supplementary Figure 10. Histone mark ablation study. (a) Violinplot shows the decrease in validation AUC when each histone mark was excluded from Chromoformer-clf training (n=11 cell types for each HM ablation experiment). Performance decreases were averaged across all the 11 cell types. Error bars denote standard error of the mean. (b) Correlation between pairs of histone mark signals. (c) Each row in the left panel shows the combination of features that were ablated simultaneously, and the corresponding row in the right panel show the decrease in AUC. The black and green boxes highlight the impact of the ablation of H3K36me3 and enhancer marks, respectively. (d) Emission probabilities of the seven histone marks for each of the 50 chromatin states inferred from chromHMM model. Similar pair of histone mark combinations were matched between panels (c) and (d). Source data are provided as a Source Data file.

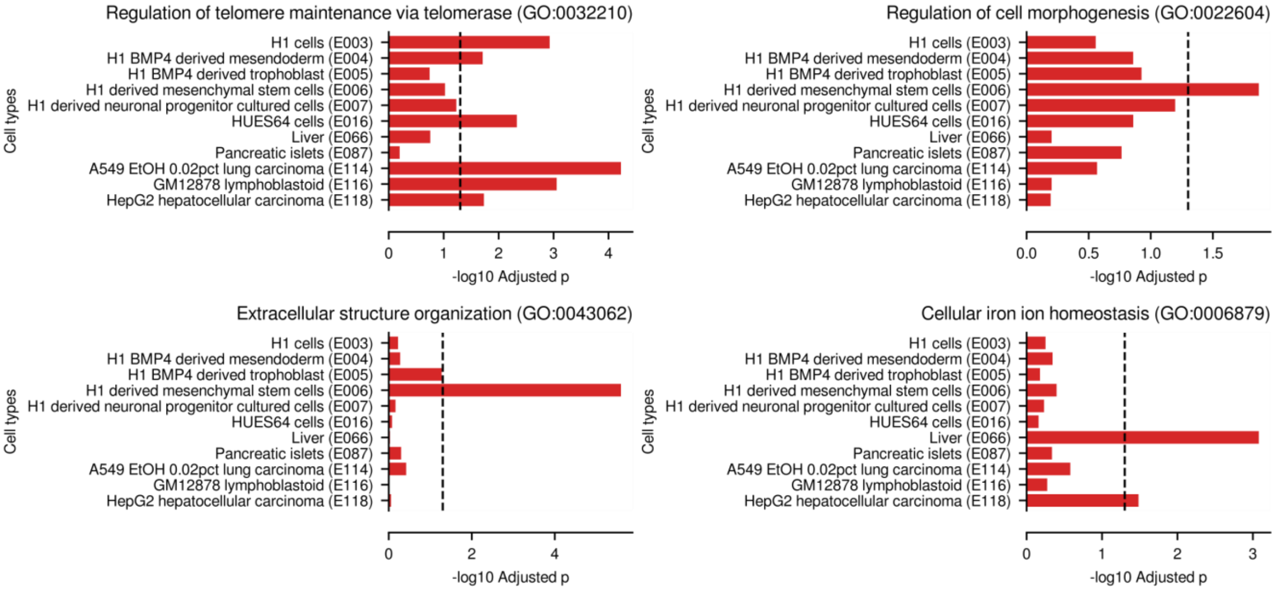

Supplementary Figure 11. Functional enrichment of highly expressed genes (i.e., expression above median) with high PCRI. For each cell type, top 250 genes with the highest PCRI values were selected for each of the four CV folds. Bars denote -log10-transformed Benjamini-Hochberg adjusted Fisher's exact p-values for the functional enrichment of the resulting 1,000 genes for each cell type.

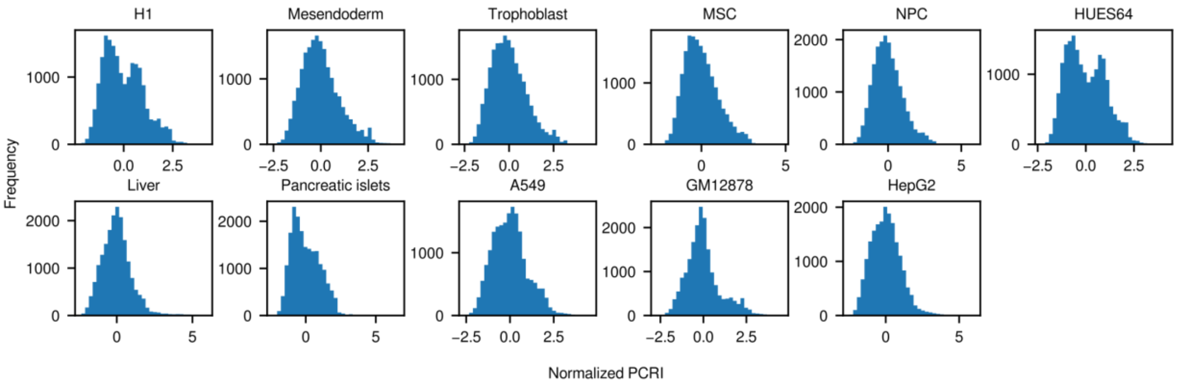

Supplementary Figure 12. Distribution of normalized PCRI values.

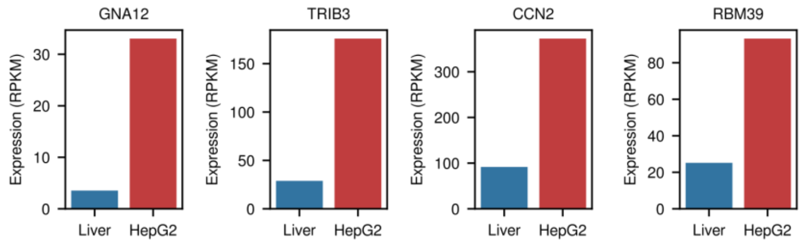

Supplementary Figure 13. Comparing the expression of *GNA12*, *TRIB3*, *CCN2* and *RBM39* in healthy liver tissue and HepG2 hepatocellular carcinoma cells. Source data are provided as a Source Data file.

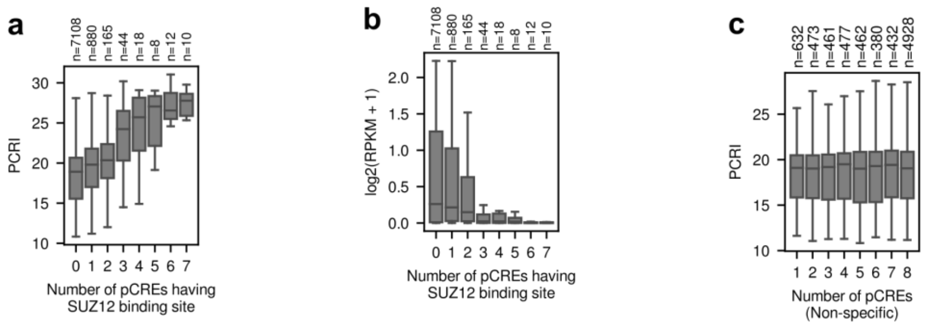

Supplementary Figure 14. Predicted effect of SUZ12-associated pCREs in *cis*-regulation learned by Chromoformer. (a) The number of pCREs harboring SUZ12 binding site versus PCRI. (b) The number of pCREs harboring SUZ12 binding site versus the actual expression level of the corresponding gene. (c) The number of non-specific pCREs versus PCRI. In the boxplot, the center line denotes the median, upper and lower box limits denote upper and lower quartiles, and whiskers denote 1.5× interquartile range. The number of genes having the corresponding number of pCREs are shown above the plot.

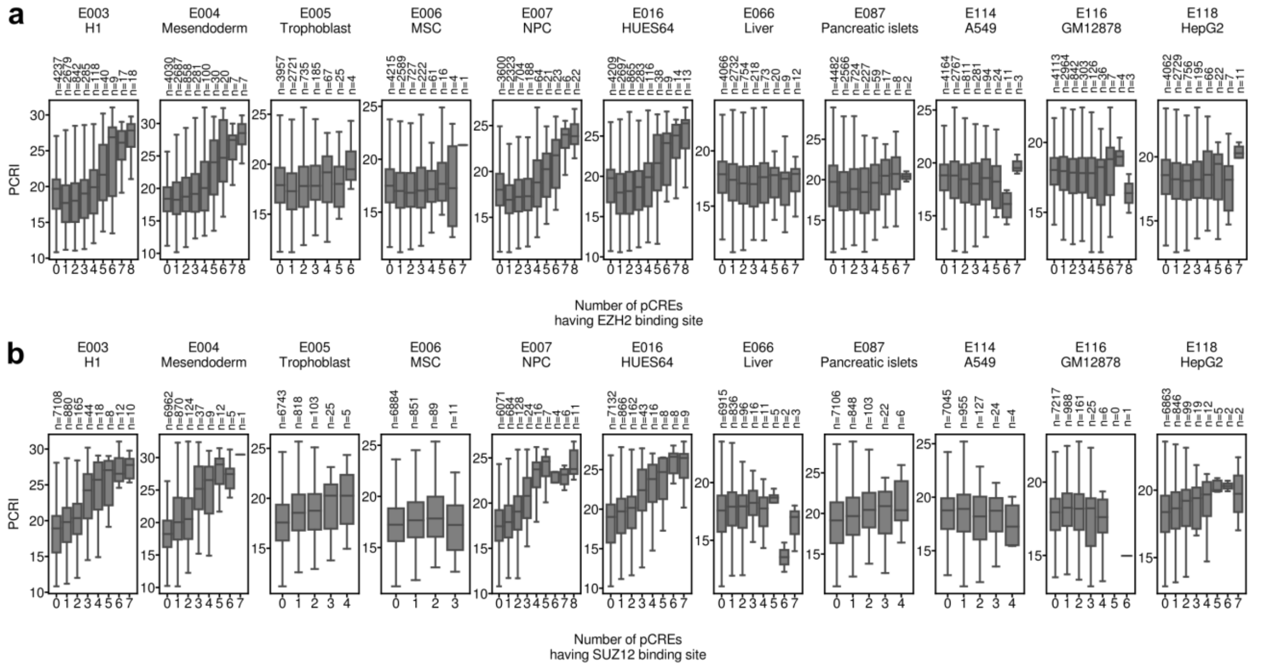

Supplementary Figure 15. Tendency of PCRI values depending on pCREs harboring PRC2 binding sites. Relationships between predicted *cis*-regulatory impact (PCRI) and the number of putative *cis*-regulatory elements (pCREs) with (a) EZH2 and (b) SUZ12 binding, which are subunits of polycomb repressive complex 2 (PRC2), are shown. In the boxplot, the center line denotes the median, upper and lower box limits denote upper and lower quartiles, and whiskers denote 1.5× interquartile range. The number of genes having the corresponding number of pCREs are shown above the plot.

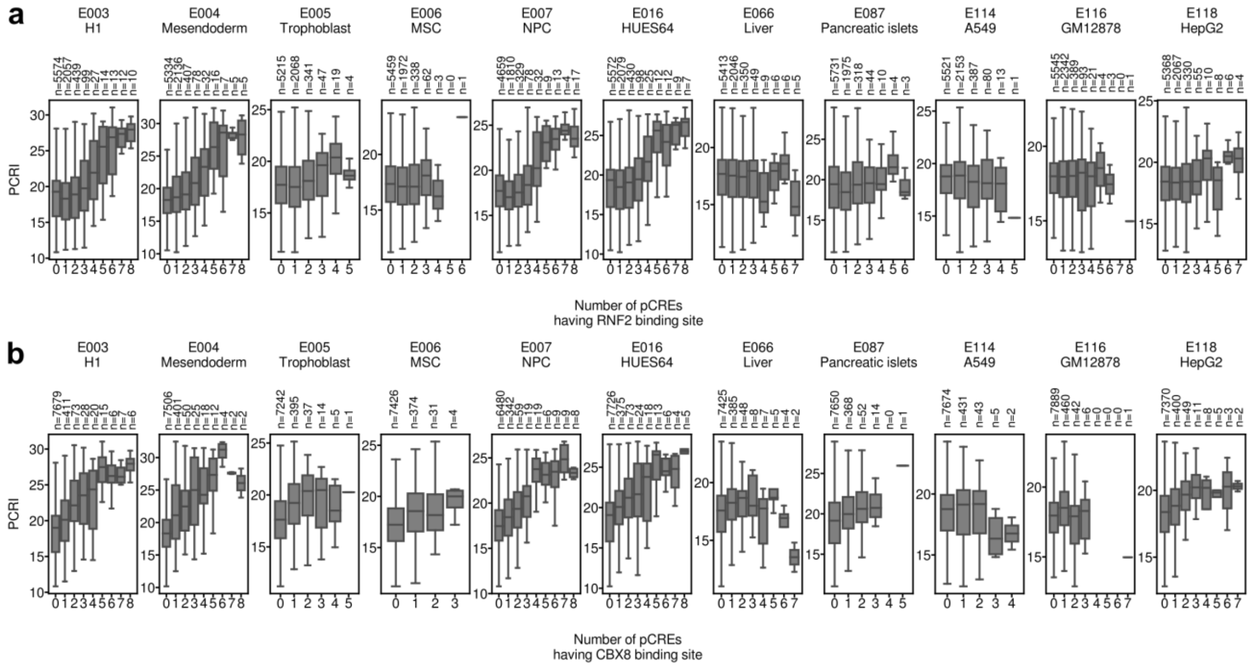

Supplementary Figure 16. Tendency of PCRI values depending on pCREs harboring PRC1 binding sites. Relationships between predicted *cis*-regulatory impact (PCRI) and the number of putative *cis*-regulatory elements (pCREs) with (a) RNF2 and (b) CBX8 binding, which are subunits of polycomb repressive complex 1 (PRC1), are shown. In the boxplot, the center line denotes the median, upper and lower box limits denote upper and lower quartiles, and whiskers denote 1.5× interquartile range. The number of genes having the corresponding number of pCREs are shown above the plot.

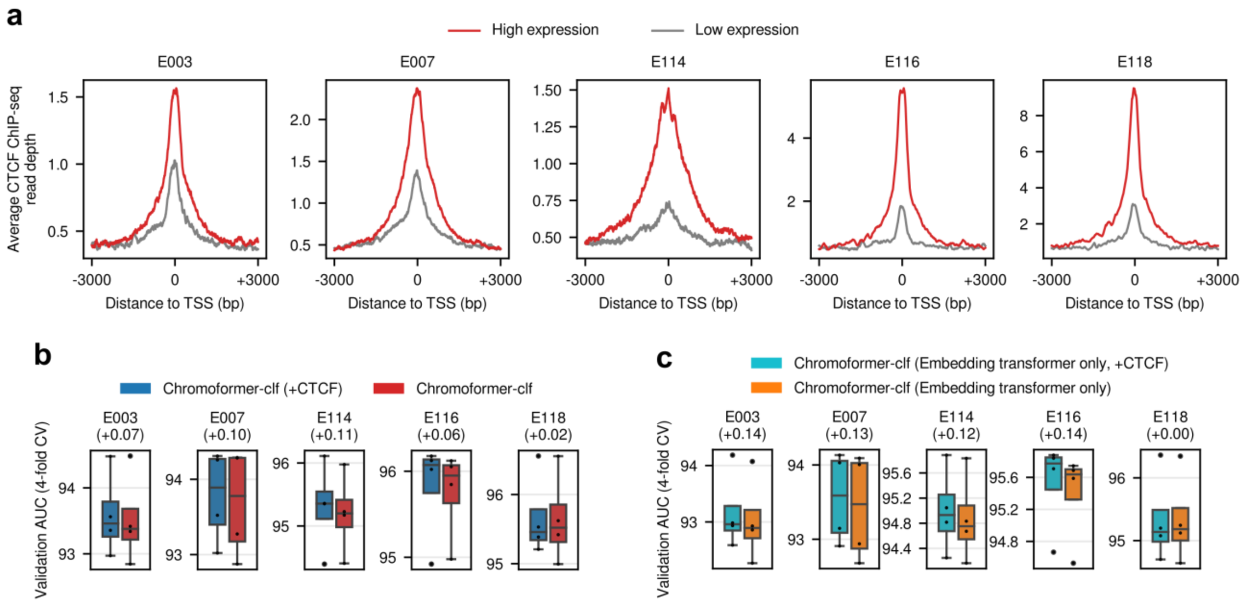

Supplementary Figure 17. Incorporating CTCF binding signals in Chromoformer training. (a) Average CTCF ChIP-seq read depths around TSS. Read depth signals were grouped and averaged based on the binary gene expression states. (b) Cross-validation (n=4) performances of Chromoformer-clf models trained with or without CTCF binding signals. (c) Cross-validation (n=4) performances of Embedding transformer-only Chromoformer-clf models trained with or without CTCF binding signals. Values in parentheses denote the amount of performance improvement when CTCF binding signals were used. In the boxplot, the center line denotes the median, upper and lower box limits denote upper and lower quartiles, and whiskers denote 1.5× interquartile range. Source data are provided as a Source Data file.

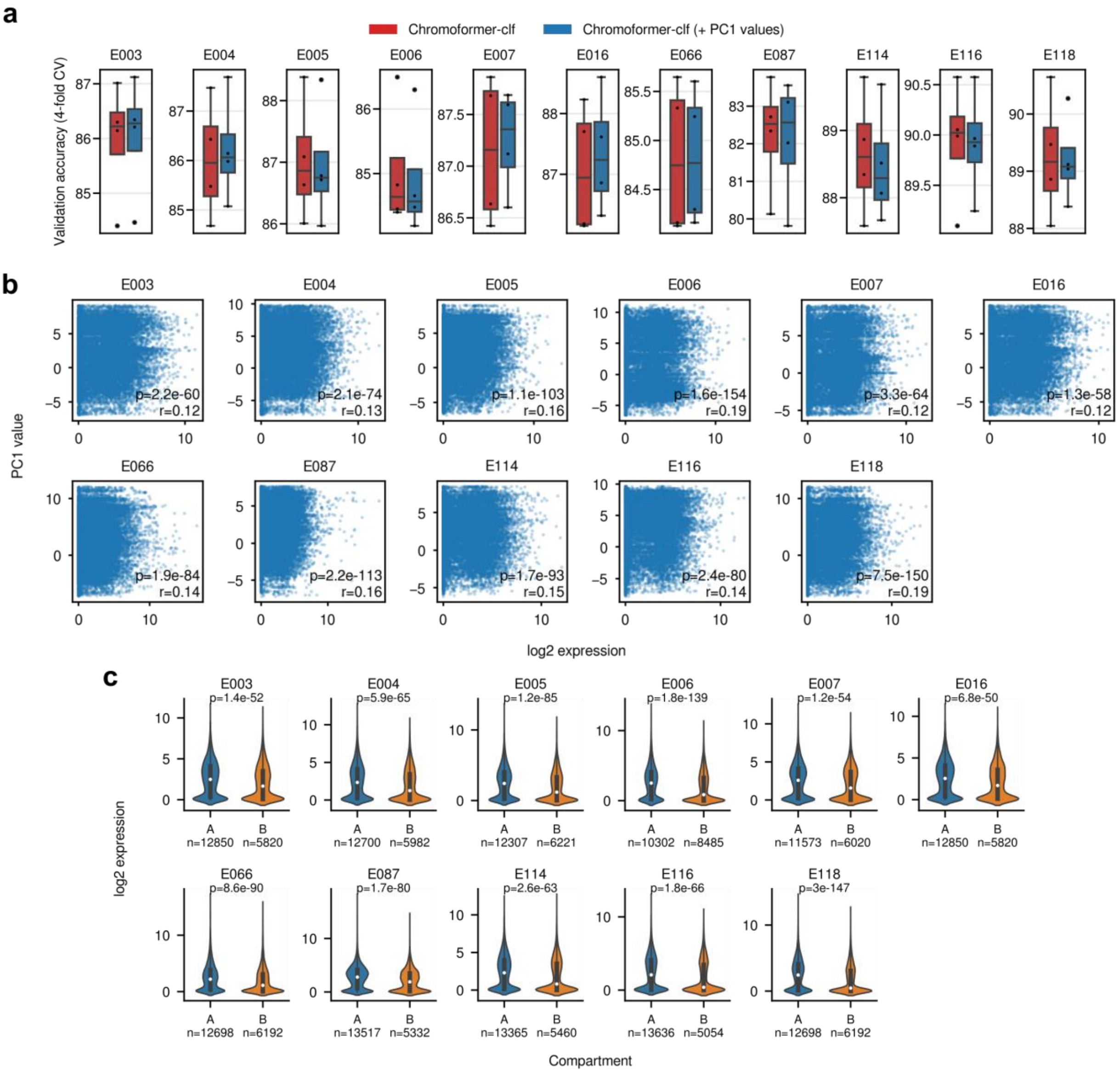

Supplementary Figure 18. Incorporating genomic compartmentalization states in Chromoformer training. (a) Cross-validation (n=4) performances of Chromoformer-clf models trained with the first principal component (PC1) values of the correlation matrix made with Hi-C contact matrix. In the boxplot, the center line denotes the median, upper and lower box limits denote upper and lower quartiles, and whiskers denote 1.5× interquartile range. (b) Pearson's correlation coefficients between gene expression and the PC1 value. P-values for the correlation coefficients are shown. (c) Distribution of gene expression based on the compartment A/B state. P-values from two-sided independent t-test are shown above, and the number of genes within each compartment are indicated below. In the boxes within the violinplot, the white point denotes the median and the upper and lower box limits denote upper and lower quartiles. Source data are provided as a Source Data file.

Supplementary references

1. Kim, K., Jung, I. covNorm: An R package for coverage based normalization of Hi-C and capture Hi-C data. *Comput Struct Biotechnol J* **19**, 3149-3159 (2021)
