# Supplementary material for: Learning the histone codes with large genomic windows and three-dimensional chromatin interactions using transformer
Source: Nat Commun. 2022 Nov 5;13:6678. doi: 10.1038/s41467-022-34152-5 (PMC9637148; doi:10.1038/s41467-022-34152-5)
Supplement: Supplementary file 3 — Reporting summary [file 41467_2022_34152_MOESM3_ESM.pdf]

nature portfolio

Corresponding author(s): Sun Kim

Last updated by author(s): Oct 13, 2022

Reporting Summary

Nature Portfolio wishes to improve the reproducibility of the work that we publish. This form provides structure for consistency and transparency in reporting. For further information on Nature Portfolio policies, see our Editorial Policies and the Editorial Policy Checklist.

Statistics

For all statistical analyses, confirm that the following items are present in the figure legend, table legend, main text, or Methods section.

n/a	Confirmed	
☐	☒	The exact sample size (*n*) for each experimental group/condition, given as a discrete number and unit of measurement
☐	☒	A statement on whether measurements were taken from distinct samples or whether the same sample was measured repeatedly
☐	☒	The statistical test(s) used AND whether they are one- or two-sided<br>*Only common tests should be described solely by name; describe more complex techniques in the Methods section.*
☒	☐	A description of all covariates tested
☐	☒	A description of any assumptions or corrections, such as tests of normality and adjustment for multiple comparisons
☐	☒	A full description of the statistical parameters including central tendency (e.g. means) or other basic estimates (e.g. regression coefficient) AND variation (e.g. standard deviation) or associated estimates of uncertainty (e.g. confidence intervals)
☐	☒	For null hypothesis testing, the test statistic (e.g. *F*, *t*, *r*) with confidence intervals, effect sizes, degrees of freedom and *P* value noted<br>*Give P values as exact values whenever suitable.*
☒	☐	For Bayesian analysis, information on the choice of priors and Markov chain Monte Carlo settings
☒	☐	For hierarchical and complex designs, identification of the appropriate level for tests and full reporting of outcomes
☐	☒	Estimates of effect sizes (e.g. Cohen's *d*, Pearson's *r*), indicating how they were calculated

Our web collection on statistics for biologists contains articles on many of the points above.

Software and code

Policy information about availability of computer code

Data collection	To collect all the required data automatically, we configured and ran a custom Snakemake (version 6.5.3) pipeline based on GNU wget, which is available at the github repository [https://github.com/dohlee/chromoformer].
Data analysis	Raw histone modification ChIP-seq alignments were processed using using bwa v0.7.17-r1188, bedtools v2.23.0, sambamba v0.6.8 and custom Python script. Deep learning models were implemented using Python 3.9 and PyTorch v1.9.0. Computation of the normalized Hi-C interaction frequencies follows the procedure of R package covNorm v1.1.0. Custom scripts for the analyses and code implementation for the deep learning model is available at the GitHub repository [https://github.com/dohlee/chromoformer]. Pretrained weights for Chromoformer-clf models are available at Figshare [https://doi.org/10.6084/m9.figshare.19424807.v1]. Code implementations for benchmark models were downloaded from the respective code repositories; DeepChrome was obtained from [https://github.com/QData/DeepChrome], AttentiveChrome was obtained from [https://github.com/QData/AttentiveChrome], DeepDiff was obtained from [https://github.com/QData/DeepDiffChrome], GC-MERGE was obtained from [https://github.com/rsinghlab/GC-MERGE] and HM-CRNN was obtained from [https://github.com/pptnz/deeply-learning-regulatory-latent-space].

For manuscripts utilizing custom algorithms or software that are central to the research but not yet described in published literature, software must be made available to editors and reviewers. We strongly encourage code deposition in a community repository (e.g. GitHub). See the Nature Portfolio guidelines for submitting code & software for further information.

Data

Policy information about availability of data

All manuscripts must include a data availability statement. This statement should provide the following information, where applicable:

- Accession codes, unique identifiers, or web links for publicly available datasets
- A description of any restrictions on data availability
- For clinical datasets or third party data, please ensure that the statement adheres to our policy

Histone ChIP-seq read alignments, mRNA expression profiles and chromHMM chromatin states used in this study were downloaded from Roadmap Epigenomics Web Portal23 [https://egg2.wustl.edu/roadmap/web_portal/index.html]. Normalized interaction frequencies for promoter-centered Hi-C experiments were obtained from hg19 pcHi-C data collection of 3DIV database [http://3div.kr/] under tissue mnemonics H1, ME, TB, MSC, NPC, LI11, PA, LG and GM. TF ChIP-seq reads targeted for PRC1/2 subunits and CTCF were also downloaded from ENCODE24 under accession codes specified in Supplementary Table 1 and 3. TAD and genomic compartmentalization information (in PC1 values) were downloaded from the Supplementary Material of Schmitt et al.43 Raw histone ChIP-seq reads and the mRNA expression profile for ES-Bruce4 mouse embryonic stem cell data were also downloaded from ENCODE24 under accession codes specified in Supplementary Table 2. Accession code for the ES-Bruce4 mRNA expression profile was ENCFF166EXS [https://www.encodeproject.org/experiments/ENCSR000CGU]. Hi-C interaction frequency matrices for ES-Bruce4 cells were downloaded from the data repository of Dixon et al.41 NCBI RefSeq gene annotations were downloaded from UCSC Table Browser [https://genome.ucsc.edu/cgi-bin/hgTables]. Gencode vM1 gene annotations were downloaded from https://www.gencodegenes.org/mouse/release_M1.html. Source data are provided with this paper.

Human research participants

Policy information about studies involving human research participants and Sex and Gender in Research.

Reporting on sex and gender	There were no human research participants in this study.
Population characteristics	There were no human research participants in this study.
Recruitment	There were no human research participants in this study.
Ethics oversight	There were no human research participants in this study.

Note that full information on the approval of the study protocol must also be provided in the manuscript.

Field-specific reporting

Please select the one below that is the best fit for your research. If you are not sure, read the appropriate sections before making your selection.

☒ Life sciences ☐ Behavioural & social sciences ☐ Ecological, evolutionary & environmental sciences

For a reference copy of the document with all sections, see nature.com/documents/nr-reporting-summary-flat.pdf

Life sciences study design

All studies must disclose on these points even when the disclosure is negative.

Sample size	No statistical tests were performed to determine sample size in this study, but the number of cell types used for model evaluation is determined based on the following criteria: A cell type should have both histone ChIP-seq and RNA-seq data in Roadmap Epigenomics Project, and have three-dimensional promoter-centered interaction data in 3DIV as well. Therefore, the number of chosen cell types (n=11) is the maximum number of different cell types that are covered by all of those multi-omics profiles required for this study (Histone modifications, RNA-seq and pcHi-C profiles).
Data exclusions	Data were not excluded from the analysis.
Replication	Performance of deep learning models were evaluated using 4-fold cross-validation. For each fold, one-fourth of the data were held out and the model was trained with the remaining data. After training the model, the model performance was evaluated using the held-out validation set. This procedures were done independently for each fold, as well as each of the 11 cell types targeted in this study.
Randomization	For 4-fold cross-validation of deep learning model performance, 18,955 protein coding genes were randomly divided into four subsets. To avoid information leakage due to the three-dimensional chromatin interactions, we forced that genes at a chromosome do not appear in two or more cross-validation folds.
Blinding	No blinded group allocation were performed since no new experimental data was collected in this study.

Reporting for specific materials, systems and methods

We require information from authors about some types of materials, experimental systems and methods used in many studies. Here, indicate whether each material, system or method listed is relevant to your study. If you are not sure if a list item applies to your research, read the appropriate section before selecting a response.

Materials & experimental systems

n/a	Involved in the study
☒	☐ Antibodies
☒	☐ Eukaryotic cell lines
☒	☐ Palaeontology and archaeology
☒	☐ Animals and other organisms
☒	☐ Clinical data
☒	☐ Dual use research of concern

Methods

n/a	Involved in the study
☒	☐ ChIP-seq
☒	☐ Flow cytometry
☒	☐ MRI-based neuroimaging
