# Supplementary material for: Learning the histone codes with large genomic windows and three-dimensional chromatin interactions using transformer
Source: Nat Commun. 2022 Nov 5;13:6678. doi: 10.1038/s41467-022-34152-5 (PMC9637148; doi:10.1038/s41467-022-34152-5)
Supplement: Supplementary file 5 — Inventory of Supporting Information [file 41467_2022_34152_MOESM5_ESM.docx]

Supplementary Note 1

Difference between Embedding transformer and Pairwise interaction transformer.

Supplementary Methods

Supplementary Table 1

ENCODE file accessions of PRC1 and PRC2 subunit ChIP-seq peaks.

Supplementary Table 2

ENCODE file accessions of raw ChIP-seq reads for ES-Bruce4 mouse embryonic stem cell.

Supplementary Table 3

ENCODE file accessions of raw CTCF ChIP-seq reads.

Supplementary Figure 1

Input feature generation procedures.

Supplementary Figure 2

Distribution of HindIII fragment length from the pcHi-C dataset used in this study.

Supplementary Figure 3

Cross-validation (n=4) performances of Chromoformer-clf models when different combinations of resolutions were used.

Supplementary Figure 4

Chromoformer-clf model performances when self-attention-based aggregation of regulatory embeddings was used instead of concatenation.

Supplementary Figure 5

Chromoformer-diff model architecture and performance.

Supplementary Figure 6

Chromoformer-clf model performance.

Supplementary Figure 7

Chromoformer-reg model performance.

Supplementary Figure 8

Contribution of inter- and intra-TAD chromatin interactions in Chromoformer training.

Supplementary Figure 9

Across-cell type consistency of self-attention weights learned by the Embedding transformer of Chromoformer-clf.

Supplementary Figure 10

Histone mark ablation study.

Supplementary Figure 11

Functional enrichment of highly expressed genes (i.e., expression above median) with high PCRI.

Supplementary Figure 12

Distribution of normalized PCRI values.

Supplementary Figure 13

Comparing the expression of *GNA12*, *TRIB3*, *CCN2* and *RBM39* in healthy liver tissue and HepG2 hepatocellular carcinoma cells.

Supplementary Figure 14

Predicted effect of SUZ12-associated pCREs in {\it cis}-regulation learned by Chromoformer.

Supplementary Figure 15

Tendency of PCRI values depending on pCREs harboring PRC1 binding sites.

Supplementary Figure 16

Tendency of PCRI values depending on pCREs harboring PRC1 binding sites.

Supplementary Figure 17

Incorporating CTCF binding signals in Chromoformer training.

Supplementary Figure 18

Incorporating genomic compartmentalization states in Chromoformer training.
